# Preparing the functional biomaterial with osteogenic bioactivities by incorporating annealing pretreated silk fiber and iron oxide nanoparticles

**DOI:** 10.3389/fbioe.2025.1584081

**Published:** 2025-04-10

**Authors:** Peng Wang, Hengda Wang, Xucai Wang, Jiayu Gu, Caoxing Huang, Jianfei Sun

**Affiliations:** ^1^ State Key Laboratory of Bioelectronics, Jiangsu Key Laboratory for Biomaterials and Devices, School of Biological Science and Medical Engineering, Southeast University, Nanjing, China; ^2^ Co-Innovation Center for Efficient Processing and Utilization of Forest Resources, College of Chemical Engineering, Nanjing Forestry University, Nanjing, China; ^3^ Jiangsu Institute of Metrology, Nanjing, China

**Keywords:** silk fiber, functional biomaterial, iron oxide nanoparticles, osteogenic differentiation, bone regeneration

## Abstract

Silk fiber (SF), a kind of bio-fiber from biomass protein fibers with biocompatibility and mechanical properties, has been widely utilized in biomedical engineering. However, SF-based bio-scaffolds often encounter challenges in promoting osteogenesis within bone tissue engineering (BTE) applications. In this study, SF-based composites were constructed via the solution casting method in the presence of IONPs (SFF_C_-IONPs), followed by annealing-induced self-assembly to form magnetic SF annealing films (SFF_CA_-IONPs). Three types of IONPs loaded SF films (SFF_CA_-50, SFF_CA_-100, and SFF_CA_-200) were prepared by altering the feeding IONPs (50 μg/mL, 100 μg/mL, and 200 μg/mL). Results demonstrated that SFF_C_ films primarily exhibited random coil structures and were water-soluble, while SFF_CA_ films demonstrated the formation of silk II structures and became water-insoluble. The incorporation of IONPs significantly enhanced the porosity, mechanical strength, and thermal stability of the SFF_CA_ films. Furthermore, the SFF_CA_-IONPs films not only exhibited excellent biocompatibility but also demonstrated enhanced osteo-inductive properties, as evidenced by increased alkaline phosphatase (ALP) activity, enhanced mineralized nodule formation, and upregulation of osteogenic gene expression. This study presents a promising bio-based material with significant potential for use as a scaffold in BTE applications.

## 1 Introduction

Bone tissue exhibits a remarkable intrinsic ability to self-heal following injury, with most damaged areas capable of regenerating their original structure and strength autonomously (Wang P. et al., 2024; Liu et al., 2022). However, when the defect exceeds a critical size threshold, this natural healing process becomes insufficient, requiring clinical intervention. Autologous and allogeneic bone grafts are commonly employed for the treatment of large bone defects (Reizabal et al., 2023). Despite their widespread use, these methods are hindered by several limitations, including the scarcity of donor tissue and relatively low success rates in transplantation (Hu et al., 2020). As a result, various natural and synthetic biomaterials have been developed for bone tissue engineering to overcome these challenges and improve the effectiveness of bone repair.

Recently, naturally derived polymer biomaterials, including silk fiber (SF), chitosan, alginic acid, and gelatin, have opened new frontiers in BTE due to their excellent biocompatibility and biodegradability (Wang P. et al., 2024; Liu et al., 2022). Among these, SF, a natural polymer fiber secreted by the silk glands of *Bombyx mori* cocoons, stands out for its remarkable biological activity. SF accounts for approximately 70%–75% of the silk protein and consists of light and heavy chains linked by a single disulfide bond (Reizabal et al., 2023). With its superior biocompatibility and adjustable structural properties, SF promotes normal cell proliferation and maintains cell viability without significant side effects (Hu et al., 2020). Furthermore, SF has been approved for clinical use in medical devices, such as absorbable sutures, surgical stents, and wound dressings (Holland et al., 2019). Recently, SF has garnered substantial attention from researchers worldwide and has been processed into a variety of matrix forms, including sponges, electrospun fibers, membranes, and hydrogels (dos Santos et al., 2024; Pan et al., 2024; Chen et al., 2020). Among these, SF-based membranes have emerged as promising biomaterials for a wide range of biomedical applications, particularly in bone tissue regeneration (Chen et al., 2024). SF-based membranes are typically fabricated using the solution casting method, which involves obtaining thin film materials through evaporation or solvent volatilization (Kramar et al., 2023). However, films obtained by this method are generally water-soluble and possess an amorphous structure, limiting their further application. To address this limitation, solvent annealing—a method that induces self-assembly of materials through organic reagents—is often combined with solution casting to produce SF films with enhanced mechanical properties and a silk II crystalline structure (Wang H. et al., 2024). Nonetheless, the relatively poor osteogenic activity of SF has restricted its full potential in bone tissue engineering applications.

One notable advantage of solvent annealing-induced self-assembly in SF composites is its versatility in incorporating a wide range of bioactive agents, including functional nanoparticles, drugs, and small molecules (Guan et al., 2020). Among these, magnetic iron oxide nanoparticles (IONPs) have gained increasing attention for their broad applicability in various biomedical fields, owing to their excellent biocompatibility, *in vivo* stability, and unique electromagnetic properties (Wang et al., 2020; Wang et al., 2025). Specifically, IONPs have shown promising osteogenic activity, with the ability to effectively promote the differentiation of stem cells into osteoblasts, making them particularly useful in the treatment of significant bone defects (Yu et al., 2020). The capacity of IONPs to interact with the biological environment and elicit favorable cellular responses, particularly by promoting osteogenic differentiation, has garnered significant attention for their potential to enhance bone tissue regeneration (Wang et al., 2016; Wang et al., 2017). Our previous study demonstrated that alendronate sodium-modified IONPs exert a synergistic therapeutic effect in bone tissue by not only regulating bone metabolism but also modulating the bone microenvironment, thereby enhancing the efficacy of osteoporosis treatment (Zheng et al., 2022). Furthermore, their magnetic properties enable precise targeting and localized treatment, offering significant advantages in therapeutic applications (Zheng et al., 2022). Till now, to the best of our knowledge, the effects of IONPs incorporated into SF matrices constructed via solvent annealing-induced self-assembly have not been extensively explored.

**FIGURE 1:**
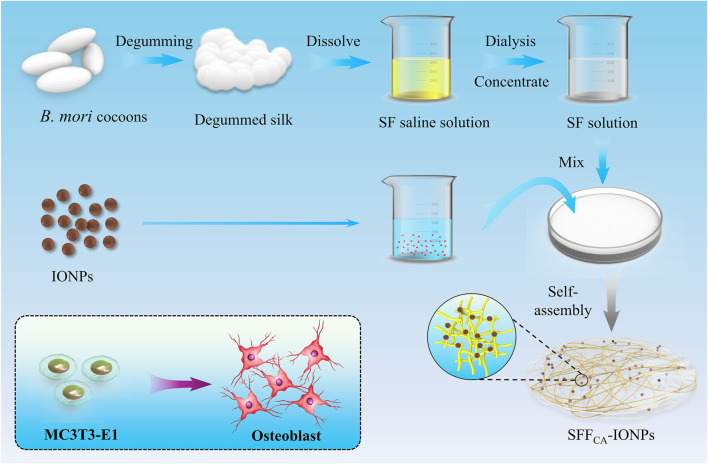
Schematic illustration of solvent annealing-induced self-assembly-based SFF_CA_-IONPs to promote osteogenic differentiation *in vitro*.

In this study, the SF-based composites were initially prepared via the solution casting method with varying concentrations of IONPs at 0, 50, 100, and 200 μg/mL, designated as SFFC series. These precursor composites were subsequently subjected to solvent annealing-induced self-assembly to produce structurally modified materials, which were correspondingly labeled as SFF_CA_-0, SFF_CA_-50, SFF_CA_-100, and SFF_CA_-200. The morphological and structural properties of both precursor and annealed composites were systematically investigated through comprehensive characterization techniques. To evaluate their biological performance, *in vitro* biocompatibility and osteogenic activity of the SFF_CA_-IONPs composites were assessed using the mouse calvarial pre-osteoblast cell line MC3T3-E1. The results demonstrated that the incorporation of IONPs into the SFF_CA_ composites significantly enhanced their osteogenic potential, promoting the differentiation of osteoblast-like cells. This study highlights the promising potential of SF-based biomaterials, particularly SF-IONP composites, in bone tissue engineering (Figure 1).

## 2 Materials and methods

### 2.1 Materials

*Bombyx mori* cocoons were generously provided by the Sericulture Institute of Soochow University (Suzhou, China). Neutral soap is purchased from Shanghai Soap Co., Ltd. (Shanghai, China). The dialysis membranes were purchased from Viskase Co., Ltd. (United States). Calcium chloride and absolute ethanol were analytically pure reagents produced by Sinopharm Chemical Reagent Co., Ltd. (Shanghai, China). The water used for all experiments was ultrapure (UP) water with resistivity above 18.2 MΩ cm.

### 2.2 Preparation of IONPs

IONPs were prepared according to a chemical co-precipitation method mentioned in the published literature (Chen et al., 2016). Briefly, 200 mg of polysaccharide was initially dissolved in 10 mL of UP water, which was thoroughly stirred to ensure uniform mixing in the presence of Nitrogen. After that, 60 mg FeCl_3_·6H_2_O and 30 mg FeCl_2_·4H_2_O were carefully added to the solution, and continuous stirring at 600 rpm was maintained for 10 min, then quickly adding 1 mL of ammonium hydroxide. The solution was heated to 80°C and held for another 30 min. Finally, the as-prepared IONPs were collected by an ultrafiltration centrifuge tube (30 kDa), washed with UP water three times, and stored at 4°C.

### 2.3 Preparation of regenerated SF solution

20 g cocoons were added to 1 L water, boiled for 60 min, and repeated once. Then, the cocoons were added to 1 L neutral soap aqueous solution with 0.2% concentration, boiled for 60 min, and thoroughly cleaned with UP water to remove silk sericin. Then, the dried silk fiber was obtained by removing moisture in an oven at 50°C. Finally, silk fiber was added to a ternary solvent, dialyzed with UP water for 48–72 h, and regenerated SF solution was obtained through removing insoluble matter by centrifugation.

### 2.4 Preparation of SFF_CA_

SFF_C_ was prepared by solution casting; the SF solution was added to the culture dishes and slowly dried at room temperature for 12 h to obtain dried composites. After that, SFF_C_ was extracted from the culture dishes and soaked in ethanol with a concentration of 90% for 30 min to form a water-insoluble SF annealing film (SFF_CA_). Solvent annealing-induced self-assembly of SF molecular chains developed a water-insoluble silk Ⅱ structure. During this process, various concentrations of IONPs (0 μg/mL, 50 μg/mL, 100 μg/mL, 200 μg/mL) were added into SFF_CA_.

### 2.5 Characterization

Transmission electron microscopy was used to analyze the morphology of IONPs (TEM, JEOL 1200EX). The hydrodynamic dimensions of IONPs were determined by dynamic light scattering (DLS, Malvern Zetasizer Nano ZS90, United Kingdom). The X-ray diffractometer (XRD, Rigaku, Japan) was used to examine the crystal structure of IONPs. The atomic force microscopy (AFM, Bruker, Germany) and field emission scanning electron microscopy (FE-SEM, Zeiss Ultra Plus, Germany) were used to characterize the morphology of SFF_C_-IONPs and SFF_CA_-IONPs. Fourier transforms infrared spectrometer (FTIR, Thermo Fisher, United States), X-ray diffractometer (XRD, Rigaku, Japan), and Raman spectrometer (Raman, Horiba Jobin Yvon, France) were used to characterize the structure of SFF_C_-IONPs and SFF_CA_-IONPs. The simultaneous thermal analyzer (TG-DSC, TA, United States) was used to analyze the thermal properties of SFF_C_-IONPs and SFF_CA_-IONPs. A Trapezium X-type tester (Shimadzu Corporation, Japan) was used to evaluate the mechanical properties of SFF_CA_-IONPs. A contact angle analyzer (Attention Theta, Biolin Scientific, Inc. Stockholm, Sweden) was used to test the dynamic water contact angle (WCA) of SFF_C_-IONPs and SFF_CA_-IONPs by the stemless drop method. The SFF_CA_-IONPs were put in PBS solution (37°C) to study their swelling properties and the release of IONPs.

### 2.6 *In vitro* experiments

#### 2.6.1 Cytotoxicity assay

The cytotoxicity of the extract was evaluated using MC3T3-E1 cells, *which were considered as an immortalized pre-osteoblast cell line that are widely used for biosafety evaluation due to their ease of culture and manipulation* (Zhang et al., 2021a). The cells were purchased from the Cyagen Biosciences Inc. (Suzhou, China). Each variant of SFF_CA_-IONPs was subjected to incubation in α-MEM medium, supplemented with fetal bovine serum (10%) and penicillin-streptomycin (1%), under standard cell culture conditions of 37°C, 5% CO_2_, and 95% humidity for 24 h to acquire the extracts. According to ISO 10993-5, specimen area to solution volume ratio was 1.25 cm^2^/mL. The extracts were filtered with 0.22 μm membrane and sterilized before use. MC3T3-E1 cells were seeded at a density of 5 × 10^3^cells/well in a 96-well plate. The cells were cultured at 37°C, 5% CO_2_, and 95% humidity for 1 day with a regular medium, and the medium was changed to different extracts for each group. After a 24-h incubation period, the cells were washed with PBS three times. Subsequently, 100 μL of regular medium containing 10% Cell Counting Kit-8 (CCK-8; Bimake, China), was added for each well, and the plates were incubated for 1 h. The absorbance at 450 nm for the solution was measured, and the relative growth rate (RGR) of the experimental groups (SFF_CA_-0, SFF_CA_-50, SFF_CA_-100, SFF_CA_-200) was calculated in comparison to the control group (Blank). In the live/dead staining procedure, cells were cultured with different extracts medium (SFF_CA_-0, SFF_CA_-50, SFF_CA_-100, SFF_CA_-200) for 24 h, followed by three washes with PBS. Subsequently, cells were stained using calcein-AM/PI (Solarbio Science and Technology Co., Ltd., China) as per the manufacturer’s instructions. The stained cells were then visualized using a laser scanning confocal microscope (Olympus 141 FV3000, Tokyo, Japan). In the phalloidin/DAPI staining process, the cell incubation step mirrored that of the live/dead staining procedure. In the subsequent step, cells were fixed with 4% paraformaldehyde, treated with 0.1% Triton X-100, and stained with phalloidin (Actin-Tracker Green-488, Beyotime Institute of Biotechnology, China) and DAPI (Thermo Fisher Scientific, United States) according to the manufacturer’s protocol. The resulting stained cells were examined using a laser scanning confocal microscope (Olympus 141 FV3000, Tokyo, Japan).

#### 2.6.2 Osteogenic differentiation

Bone marrow-derived mesenchymal stem cells (rBMSCs) were chosen for osteogenic performance validation as they more closely resemble the physiological process of bone formation compared to the MC3T3-E1 cell line, thereby offering a more precise and biologically relevant assessment of the material’s impact on osteogenesis (Zhang et al., 2021b). rBMSCs were seeded at 1 × 10^5^ cells/mL in 24-well plates at 37°C in 5% CO_2_ for 14 and 28 days, respectively. After 1-day incubation, the culture medium was replaced with sample extracts supplemented with 50 mM ascorbic acid, 10 mM β-glycerophosphate, and 100 nM dexamethasone (Sigma-Aldrich, United States). The supplemented extracts were refreshed every 2 days. After 14 days of incubation, ALP staining was performed using the BCIP/NBT Alkaline Phosphatase Color Development kit (Beyotime Institute of Biotechnology, China) according to the manufacturer’s protocol. The cultured cells were washed three times with PBS and fixed with 4% paraformaldehyde (Solarbio Science and Technology Co., Ltd., China) for 15 min and washed again in PBS. The fixed cells were soaked in BCIP/NBT solution for 30 min at room temperature, washed with PBS, and were then observed under a digital camera. After induction for 28 days, alizarin red staining was used to analyze the formation of calcium nodules (mineralization). In brief, the adherent cells were fixed in 4% paraformaldehyde (Solarbio Science and Technology Co., Ltd., China) for 10 min, stained with 2%, pH = 4.2 alizarin red solution (Beyotime Institute of Biotechnology, China) for 30 min, and rinsed with phosphate buffer saline (PBS) to eliminate non-specific staining, images were captured under a digital camera.

The MC3T3-E1 cells were cultured in different experimental groups supplemented with osteogenic factors for 3 days, as previously described. Total protein extraction from MC3T3-E1 cells subjected to various treatments was performed using RIPA lysis buffer (Sigma-Aldrich, United States) supplemented with 1 mM phosphatase inhibitor cocktail and 1 mM phenylmethanesulfonyl-fluoride (Sigma-Aldrich, United States). Western blot analysis was carried out following established protocols. The primary antibodies included ALP, RUNX2, and β-Actin, which were sourced from ABclonal Biotechnology Co., Ltd. (United States). Following incubation with horseradish peroxidase-conjugated goat anti-rabbit/mouse secondary antibodies (Biosharp Technology Co., Ltd., China), western blots were visualized using the ChemiDocXRS + Imaging System. Quantitative protein analysis was performed using ImageJ (version 1.8.0). All antibodies utilized in this study were commercially sourced.

Cellular mRNA was extracted using the RNA-quick Purification Kit, and quantitative polymerase chain reaction (qPCR) was conducted with the SYBR Green Q-PCR Kit (Vazyme, Nanjing, China) on a Light Cycler 480 PCR System (Roche, Switzerland). The primer sequences utilized are provided in the Supplementary Table S1.

### 2.7 Statistical analysis

The data were analyzed using OriginPro 2024 (Learning edition) software. The results are reported as mean ± SD. Analysis of variance (ANOVA) was used to evaluate the differences between groups (*P < 0.05; **P < 0.01; ***P < 0.001).

## 3 Results and discussion

### 3.1 The characterization of IONPs

**FIGURE 2:**
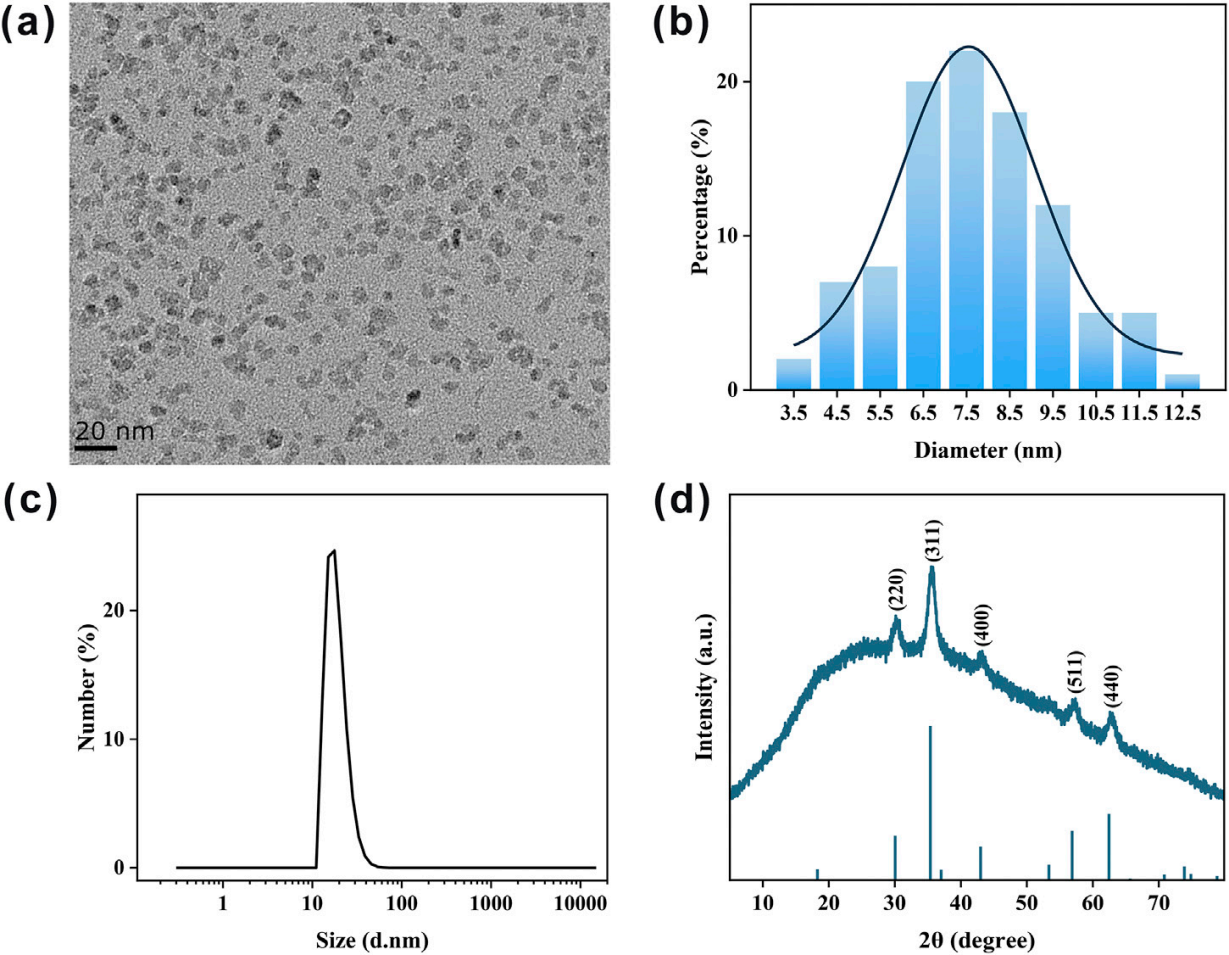
Characterization of as-prepared IONPs. **(a)** Typical TEM image of IONPs. **(b)** Statistical size distribution of IONPs in **(a)**. **(c)** DLS measurement of synthesized IONPs. **(d)** XRD pattern of IONPs.

IONPs were synthesized via a chemical co-precipitation method, utilizing polyglucose-sorbitol-carboxymethyl ether, a polysaccharide, as a stabilizer to ensure optimal dispersibility and biocompatibility (Chen et al., 2016). The morphology of the IONPs is shown in Figure 2a, where they are coated with polysaccharides and exhibit an approximately spherical shape with excellent mono-dispersity. Based on TEM analysis, the average particle size of the IONPs is measured to be 7.68 ± 1.89 nm (Figure 2b). DLS measurements reveal that the hydrodynamic size of the IONPs is 18.98 ± 5.41 nm (Figure 2c), which is larger than the physical size due to the presence of an external hydration shell surrounding the nanoparticles (Bao et al., 2023). The polydispersity index (PDI) of the IONPs is 0.38, indicating their excellent dispersibility in solutions. Furthermore, the IONPs exhibit a Zeta potential of −12.81 ± 1.62 mV, attributed to the negative charge provided by the carboxyl groups present on the polysaccharide coating. X-ray diffraction (XRD) analysis (Figure 2d) shows distinct peaks at the crystal planes (220), (311), (400), (511), and (440), which correspond to the typical diffraction patterns of Fe_3_O_4_, confirming that the IONPs synthesized in this study possess well-defined crystalline structures.

### 3.2 The surface topography of SFF_C_-IONPs and SFF_CA_-IONPs

**FIGURE 3:**
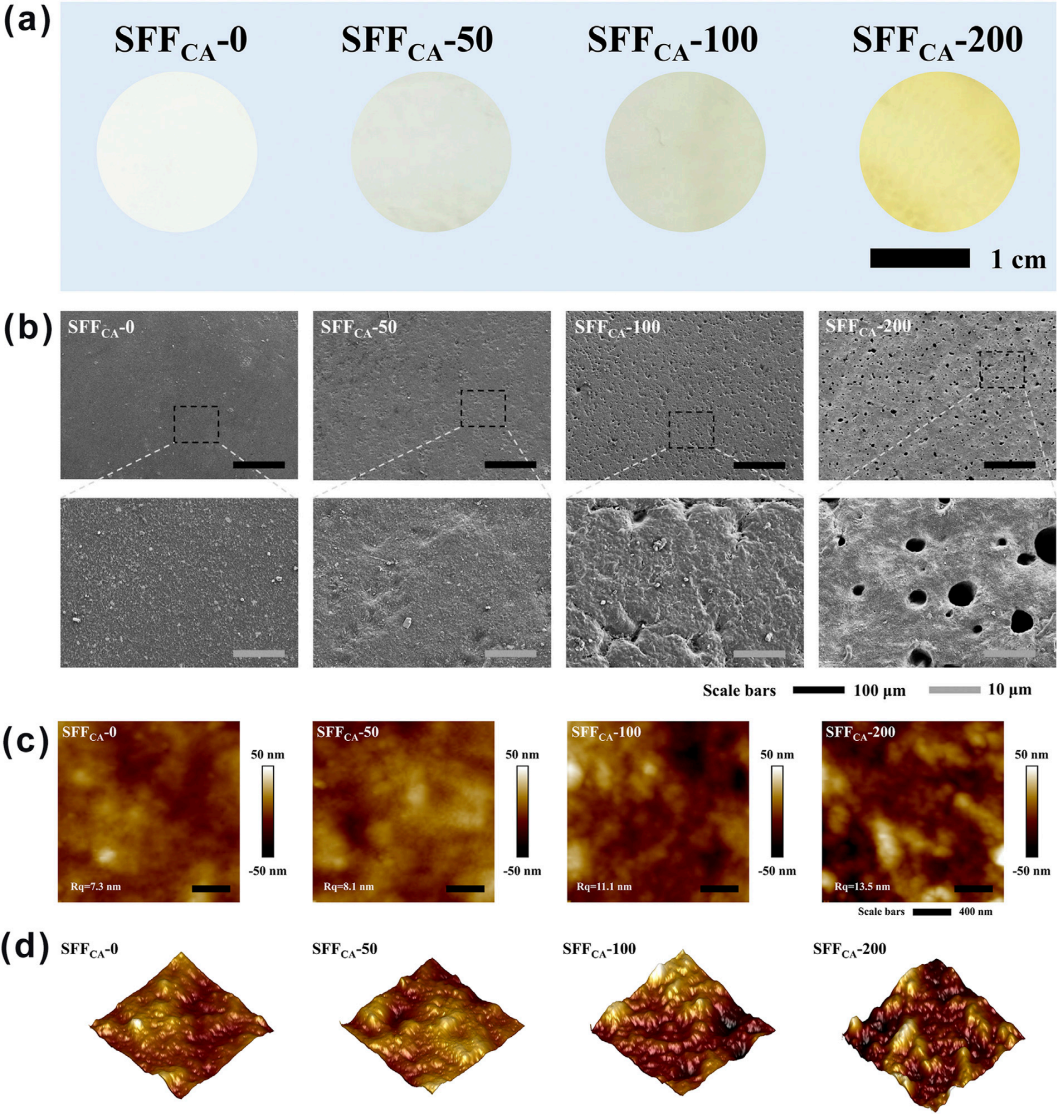
Surface topography of SFF_CA_-IONPs. **(a)** Optical images of SFF_CA_-IONPs, from left to right: SFF_CA_-0, SFF_CA_-50, SFF_CA_-100, SFF_CA_-200. **(b)** SEM images, **(c)** 2D and **(d)** 3D AFM images of SFF_CA_-IONPs.

Silk, a natural fibrous protein produced by silkworms, has garnered significant attention as a promising polymer material for membrane fabrication in the context of tissue engineering due to its remarkable biocompatibility and potential for further applications (Wang H.-Y. et al., 2024). In this study, high-molecular-weight SF, obtained through neutral soap degumming, was employed to prepare the composite. As shown in Figure 3a, the SFF_CA_-IONPs composite exhibits good uniformity, with the transparency of the composites progressively decreasing as the concentration of IONPs increases. This decrease in transparency suggests that the IONPs are uniformly dispersed throughout the film matrix. SEM images (Figure 3b) reveal that the surface of SFF_CA_-0 is smooth and devoid of pores. However, with the incorporation of higher concentrations of IONPs, small pores gradually appear on the surface of the composite. These pores may enhance cell adhesion and migration, which are critical for the applications of bone tissue regeneration (Perez and Mestres, 2016). Additionally, the surface morphology of SFF_CA_-IONPs was further examined using AFM. Figures 3c,d show the 2D and 3D AFM images of the composite, respectively. The contrast between light and dark areas in the images corresponds to the raised and recessed regions on the surface, respectively. The AFM analysis indicates that the composites maintain a relatively flat surface with an average roughness of approximately 10 nm, suggesting that the incorporation of IONPs does not result in significant alterations to the surface texture of the composites.

### 3.3 The structural properties of SFF_C_-IONPs and SFF_CA_-IONPs

Annealing treatment is a heat treatment process in which metal materials are exposed to high temperatures for a prolonged period and subsequently cooled at a controlled rate. The primary objectives of this process are to relieve internal stresses, enhance the ductility and toughness of the material, and facilitate the formation of specific microstructures. Similarly, regenerated SF solutions can be processed into various SF-based biomaterials, including composites, fibers, gels, and particles, with improved water stability, mechanical strength, or elasticity. These enhancements necessitate the application of post-processing techniques, commonly referred to as annealing (Johari et al., 2020). This annealing process is a critical step in the development and preparation of new SF biomaterials, as it governs the structural transformation of regenerated SF. The term “annealing” in the context of SF was first introduced by Tsukada, who demonstrated that exposure to methanol concentrations greater than 80% could induce a significant structural transition in SF composites (Tsukada et al., 1998). Conversely, treatment with aqueous methanol solutions below 40% could result in partial annealing of the film. The molecular structure of SF consists of a combination of random coils, α-helices, β-sheets, and β-turns, with the reverse parallel β-sheet being the defining feature of the silk II crystal structure. When regenerated SF solutions are dehydrated and dried to form specific shapes, such as SF membranes, their structure is typically dominated by random coil formations (Asakura et al., 1984). However, annealing treatments can alter the secondary and tertiary structures of SF, allowing for the fine-tuning of material properties to suit various biomedical applications. In this study, the structural transformation of SF-based composites containing IONPs (SFF_CA_-IONPs) was induced through solvent annealing. A detailed investigation of the structural changes induced by this annealing process is essential to fully understand the resulting material properties and their potential for BTE applications.

**FIGURE 4:**
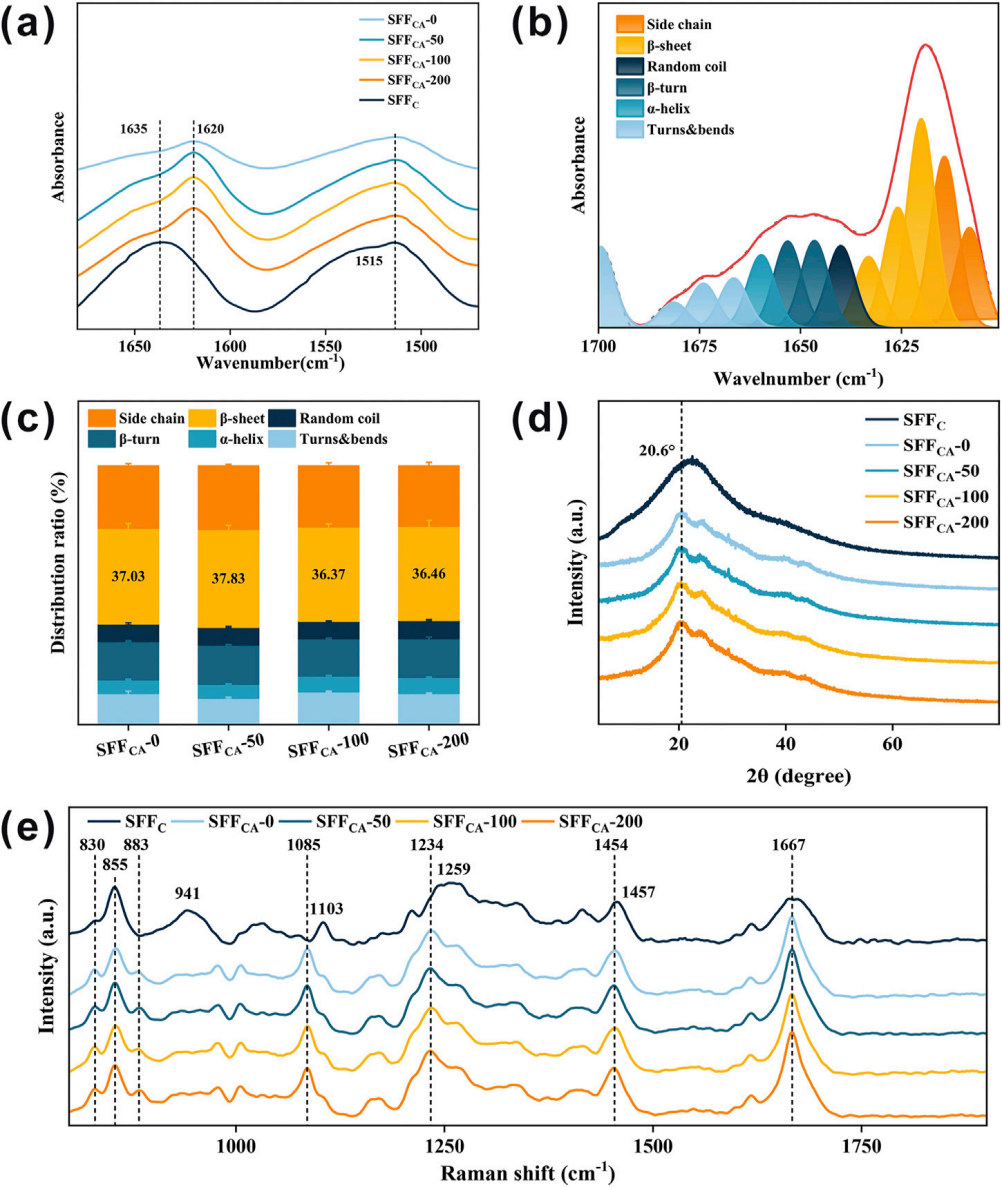
Structural properties of SFF_C_-IONPs and SFF_CA_-IONPs. **(a)** FTIR spectra of SFF_C_-IONPs and SFF_CA_-IONPs. Relative proportions of **(b)** amino acid secondary structures and **(c)** β-sheet in SFF_CA_-IONPs. **(d)** XRD pattern, and **(e)** Raman spectrum of SFF_C_-IONPs and SFF_CA_-IONPs.

FTIR is a widely employed technique for characterizing the secondary structure of SF (Cebe et al., 2017). In the infrared spectrum, the range of 1,700–1,500 cm^−1^ corresponds to absorption by the polypeptide backbone of SF molecules, encompassing the amide I (1,700–1,600 cm^−1^) and amide II (1,600–1,500 cm^−1^) bands. As shown in Figure 4a, the solution-cast SF composites (SFF_C_) exhibit a distinct absorption peak near 1,635 cm^−1^, which is characteristic of a random coil conformation, suggesting that the SFF_C_ composites possess an amorphous structure. In contrast, the absorption peak of SF composites subjected to solvent annealing (SFF_CA_-IONPs) shifts to approximately 1,620 cm^−1^, corresponding to the typical absorption of β-sheet structures. This shift indicates that solvent annealing effectively induces a conformational transition from random coils to β-sheets in the SF composites. Furthermore, the varying concentrations of IONPs had no significant impact on the infrared absorption peaks of SFF_CA_-0, SFF_CA_-50, SFF_CA_-100, and SFF_CA_-200, suggesting that the presence of IONPs does not alter the secondary structure of SF. To investigate this further, the amide I band was deconvoluted into multiple peaks, each representing a different secondary structure. As illustrated in Figures 4b,c, the relative proportions of secondary structures in the amide I band were analyzed. It was observed that the β-sheet conformation accounted for a significant proportion, exceeding 30% in both cases, and the addition of IONPs did not notably affect the overall secondary structure of the SFF_CA_-IONPs composites.

XRD was employed to further investigate the crystallization of SFF_CA_-IONPs. Previous studies demonstrated that the characteristic diffraction peaks associated with the silk II crystal structure typically appear at 9.1°, 18.9°, and 20.7° (Zhao et al., 2021). As shown in Figure 4d, the SFF_C_ composites exhibit a broad peak without distinct crystallization features, indicative of an amorphous structure. In contrast, following ethanol treatment, all SFF_CA_ composites—SFF_CA_-0, SFF_CA_-50, SFF_CA_-100, and SFF_CA_-200 display sharp diffraction peaks around 20.6°, which correspond to the characteristic diffraction of the silk II crystal structure, confirming its formation. Notably, a crystallization peak around 24.3° is observed in the SFF_CA_-IONPs composites, a peak traditionally attributed to silk I in earlier studies. However, more recent studies suggest that this peak may also belong to silk II. These findings collectively demonstrate that solvent annealing with ethanol effectively induces the transition of SFF_C_ to a well-defined silk II crystal structure.

Raman spectroscopy is commonly employed in conjunction with FTIR and XRD to further verify the structural transitions of SF, as it provides valuable insights into the secondary and tertiary structures of proteins (Monti et al., 2001). As shown in Figure 4e, the significant scattering peaks observed in SFF_C_ are at 855 cm^−1^, 941 cm^−1^, 1,103 cm^−1^, 1,259 cm^−1^ (amide III), and 1,667 cm^−1^ (amide I), which are primarily influenced by the random coil conformation and tyrosine residues. Upon the formation of the silk II crystal structure in SFF_CA_-IONPs following solvent annealing with ethanol, a noticeable shift or decrease in these scattering peaks occurs. Specifically, the scattering peak at 855 cm^−1^, associated with tyrosine residues, decreases in intensity, while a shoulder peak at 830 cm^−1^ increases. As a result, the intensity ratio of 855 to 830 cm^−1^ decreases, and a new scattering peak appears near 883 cm^−1^. Additionally, the broad peak near 941 cm^−1^ vanishes, while the scattering peak at 1,103 cm^−1^ shifts to 1,085 cm^−1^, exhibiting sharper features. The broad amide III region peak at 1,259 cm^−1^ shifts to 1,234 cm^−1^, and the scattering peak at 1,457 cm^−1^ moves to approximately 1,454 cm^−1^. These changes are indicative of the silk II structure’s formation in SFF_CA_-IONPs, which is further confirmed by a lower I_850_/I_830_ intensity ratio. This ratio reduction suggests that the tyrosine residues are primarily buried within the protein structure, with their hydroxyl groups acting as significant hydrogen bond donors, similar to natural silk fibers rich in β-sheet structures. During the transition to the silk II structure, the tyrosine residues shift from a hydrophobic to a more hydrophilic environment, forming strong hydrogen bonds. The stable formation of the silk II crystal structure in SFF_CA_-IONPs is thus supported by Raman data, in conjunction with the FTIR and XRD results.

The energy landscape theory provides a compelling framework to explain the (meta) stability of SF conformations. Metastable random coils exhibit dynamic stability at the lowest local energy state, whereas stable β-sheets represent the lowest global energy state (Harrington and Fratzl, 2021). Consequently, the transition from random coils to β-sheets is nearly inevitable for SF solutions under appropriate conditions. In this study, ethanol—a hydrophilic solvent with strong dehydrating properties—was employed as the annealing solvent to promote the formation of SF materials predominantly comprising the β-sheet (silk II) structure. Ethanol can rapidly penetrate protein structures, effectively facilitating the crystallization of amorphous SF into a more thermodynamically stable silk II structure. When exposed to sufficiently concentrated ethanol aqueous solutions, SF undergoes a structural transition from random coil or silk I to silk II, driven by the solvent’s ability to disrupt interactions between water and SF molecules through proton exchange. This interaction renders regenerated SF materials water-insoluble, enhancing their stability for various applications. The results confirmed that the incorporation of varying concentrations of IONPs does not significantly alter the secondary or crystal structure of SFF_CA_-IONPs, underscoring the robustness of the SF matrix in maintaining its conformational integrity while enabling the incorporation of functional additives.

### 3.4 The structure stability SFF_CA_-IONPs

**FIGURE 5:**
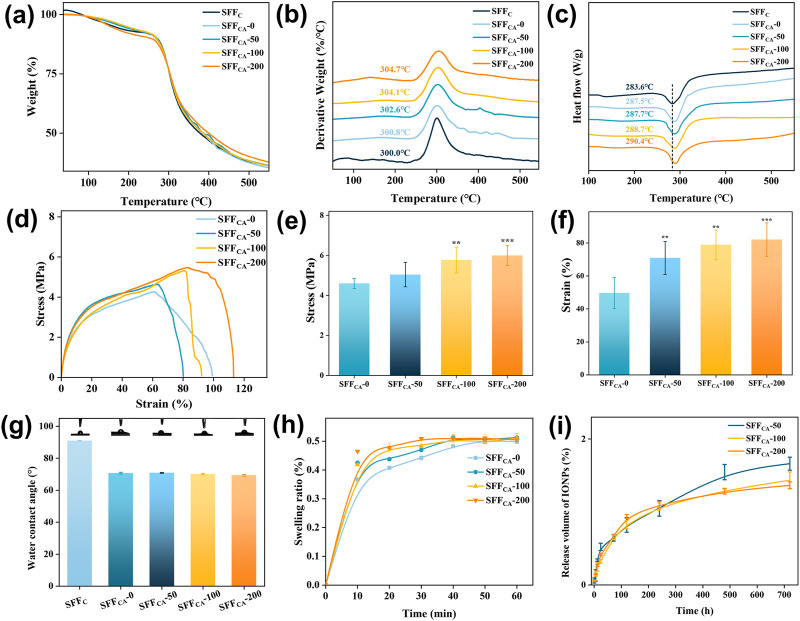
**(a–c)** TG, DTG, and DSC thermograms of SFF_C_-IONPs and SFF_CA_-IONPs. **(d)** Strain-stress curves, **(e)** Young’s modulus of SFF_CA_-IONPs. **(f)** Elongation at break of SFF_CA_-IONPs. **(g)** The water contact angle of SFF_C_-IONPs and SFF_CA_-IONPs. **(h)** Swelling ratio of SFF_CA_-IONPs. **(i)** The release of IONPs in SFF_CA_-IONPs. Asterisk indicates statistically significant differences between the control and experimental groups (**: P < 0.01; ***: P < 0.001).

To investigate the thermal stability of SFF_C_ and SFF_CA_-IONPs, thermogravimetry and differential scanning calorimetry were used to examine changes in the thermogravimetry (TG), derivative thermogravimetry (DTG), and differential scanning calorimetry (DSC) curves during heating. As shown in Figure 5a, the TG curve mainly shows three main weight loss stages. In the first stage (<100°C), the volatilization of water causes a slight decrease in SF quality. In the second stage (100°C–300°C), the loss of low-temperature volatile compounds causes the slow decomposition of SF. In the third stage (>300°C), the peptide bonds and side groups are broken, resulting in a peak in the thermal decomposition of SF. SFF_CA_-200 had the highest residual amount after the third stage of decomposition, owing to its higher IONPs concentration than other groups. As depicted in Figure 5b, the temperature was about 300°C when SFF_C_ reached the maximum decomposition rate. The temperature increased when the thermal decomposition rate of SFF_CA_-0, SFF_CA_-50, SFF_CA_-100, and SFF_CA_-200 reached the maximum, reaching 300.8°C, 302.6°C, 304.1°C, and 304.7°C respectively, indicating that the degradation temperature of silk Ⅱ structure is higher than that of random coil structure. The DSC curve showed (Figure 5c) that all the degradation peaks appear above 260°C, indicating good thermal stability. The degradation peak of SFF_C_ occurred at 283.6°C. In contrast, the degradation peak of SFF_CA_-IONPs shifted to a higher temperature, appearing at 287.5°C, 287.7°C, 288.7°C, and 290.4°C, respectively, indicating that the structure of silk Ⅱ needed to absorb more heat for degradation. The thermal analysis results showed that SFF_CA_-IONPs with silk Ⅱ structure provide improved thermal stability, and the introduced IONPs could improve the thermal stability of SFF_CA_.

Mechanical properties are a key determinant in defining the applicability of materials across various domains. In this study, SFF_CA_-IONPs were subjected to tensile testing to evaluate their mechanical performance. The stress-strain analysis (Figure 5d) revealed an increase in tensile strength from 4.6 MPa in SFF_CA_-0 to 6.0 MPa in SFF_CA_-200 (Figure 5e). Young’s modulus of SFF_CA_-IONPs remained consistent at approximately 0.8 MPa across all groups, indicating that the addition of IONPs enhanced tensile strength without significantly affecting elasticity. Furthermore, the incorporation of IONPs significantly increased their elongation at the break (Figure 5f). These findings suggest that SFF_CA_-IONPs, characterized by a silk II-dominant structure, possess promising potential for tissue engineering applications.

Surface hydrophilicity, assessed via WCA measurements, demonstrated improved wettability for SFF_CA_-IONPs, with WCA values decreasing from 90° for SFF_C_ to approximately 70° for SFF_CA_-IONPs (Figure 5g). This improvement is attributed to the transition from random coil to silk II structure, while the inclusion of IONPs did not alter the WCA further. Swelling analysis, a key indicator of polymer compactness, showed an initial rapid increase in swelling rate exceeding 30% within 10 min, plateauing at approximately 50% within 60 min, with IONP addition having negligible impact (Figure 5h).

Additionally, the release behavior of IONPs, a feature contributing to biological functionalities such as cell proliferation, was investigated. As illustrated in Figure 5i, the release profile exhibited an initial rapid phase, reaching a peak within 10 days, followed by a gradual release, culminating at 1.37% ± 0.05% after 30 days for SFF_CA_-200. The low overall release rate is attributed to the robust silk II crystalline structure of SFF_CA_-IONPs, which restricts internal material outflow. Taken together, SFF_CA_-IONPs demonstrate enhanced mechanical properties, improved hydrophilicity, and controlled IONP release, making them versatile candidates for advanced tissue engineering applications.

### 3.5 *In vitro* studies

#### 3.5.1 Biocompatibility

**FIGURE 6:**
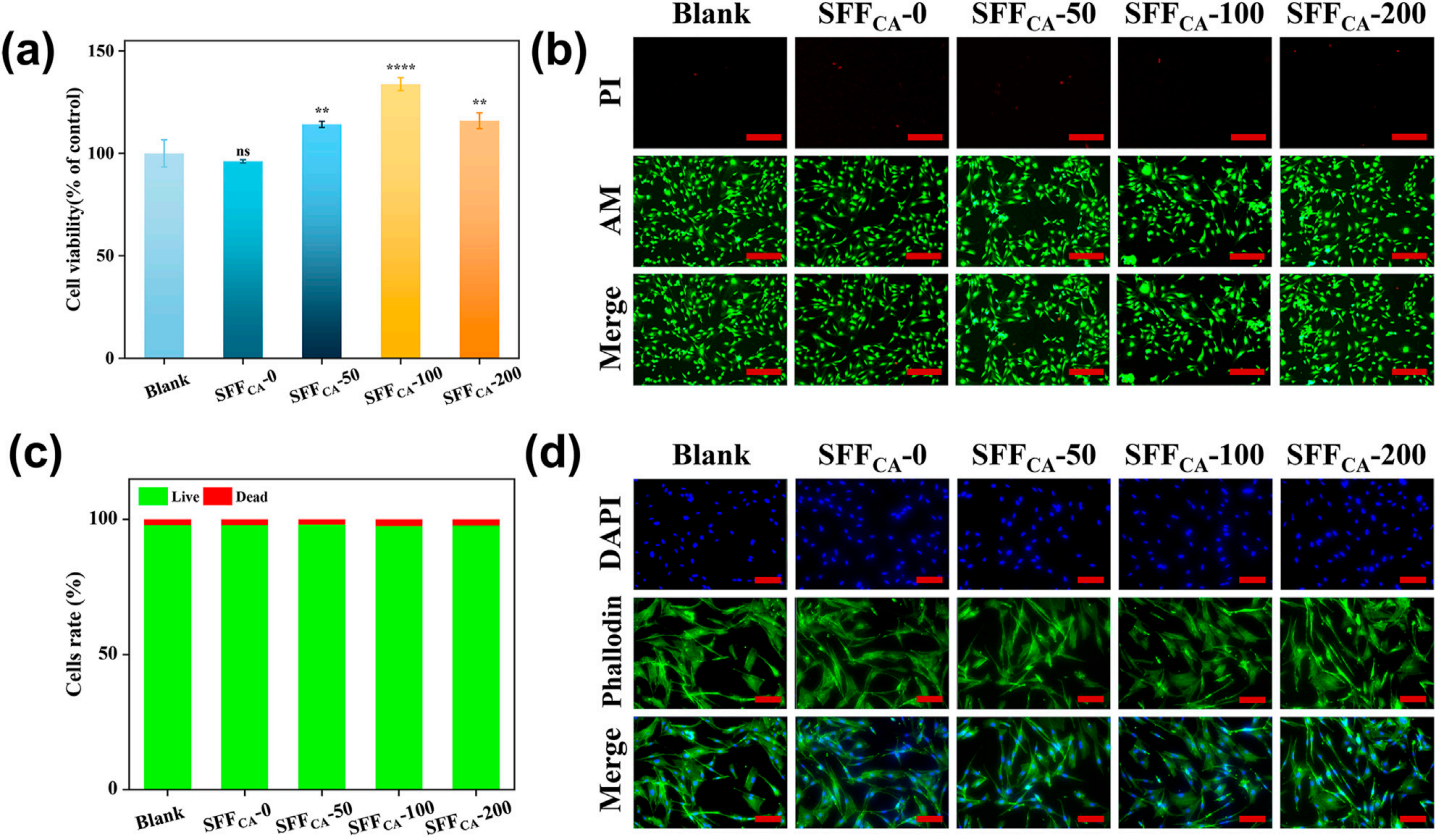
Biocompatibility of SFF_CA_-IONPs. **(a)** Cell viability of MC3T3-E1 cells after treated with the leach liquor of SF/IONPs composites for 24 h. **(b)** Representative scan of live/dead staining of MC3T3-E1 cells after treated with the leach liquor of SFF_CA_-IONPs for 24 h, green and red labeled cells represent living and dead cells, respectively **(c)** quantitative data of the live and dead cells, and **(d)** cytoskeleton photographs of SFF_CA_-IONPs. Asterisk indicates statistically significant differences between control and experimental group (**: P < 0.01; ****: P < 0.0001; ns, not significant). The scale bar represents 200 μm in **(b)**, and 100 μm in **(d)**.

The biocompatibility of materials is a critical determinant in facilitating cellular adhesion, migration, and proliferation, which are essential for the success of bone tissue engineering (Huang et al., 2023). Cell viability assessed via the CCK-8 assay demonstrated that Silk fiber composites loaded with IONPs exhibited significantly higher viability compared to the control and SFF_CA_-0 groups (Figure 6a). This enhancement in cell activity may be attributed to the gradual degradation of Silk fiber, which releases IONPs into the medium. Furthermore, the cytotoxicity of SFF_CA_-IONPs was assessed using a live/dead assay. As shown in Figures 6b,c, the majority of cells remained viable after 24 h of incubation, with no statistically significant differences in the live/dead cell ratios. These results indicated that SFF_CA_ with incorporation of various amounts of IONPs did not exhibit detectable cytotoxicity. Cell morphology analysis revealed well-spread cells with intact cytoskeletons, uniform morphologies, and distinct pseudopodia across all groups (Figure 6d). Despite prior reports suggesting potential morphological alterations at higher concentrations of IONPs, the maximum release of IONPs in SFF_CA_-200, measured at 1.37% ± 0.05% after 30 days, was substantially below the threshold of 300 μg Fe/mL identified in previous studies as inducing minor morphological changes (Soenen et al., 2011; Toropova et al., 2017). Consequently, the gradual release of low-concentration IONPs from SFF_CA_-IONPs not only supports biocompatibility but also enhances cell viability without altering cell morphology, offering promising potential for bone tissue engineering applications.

#### 3.5.2 Osteogenesis

**FIGURE 7:**
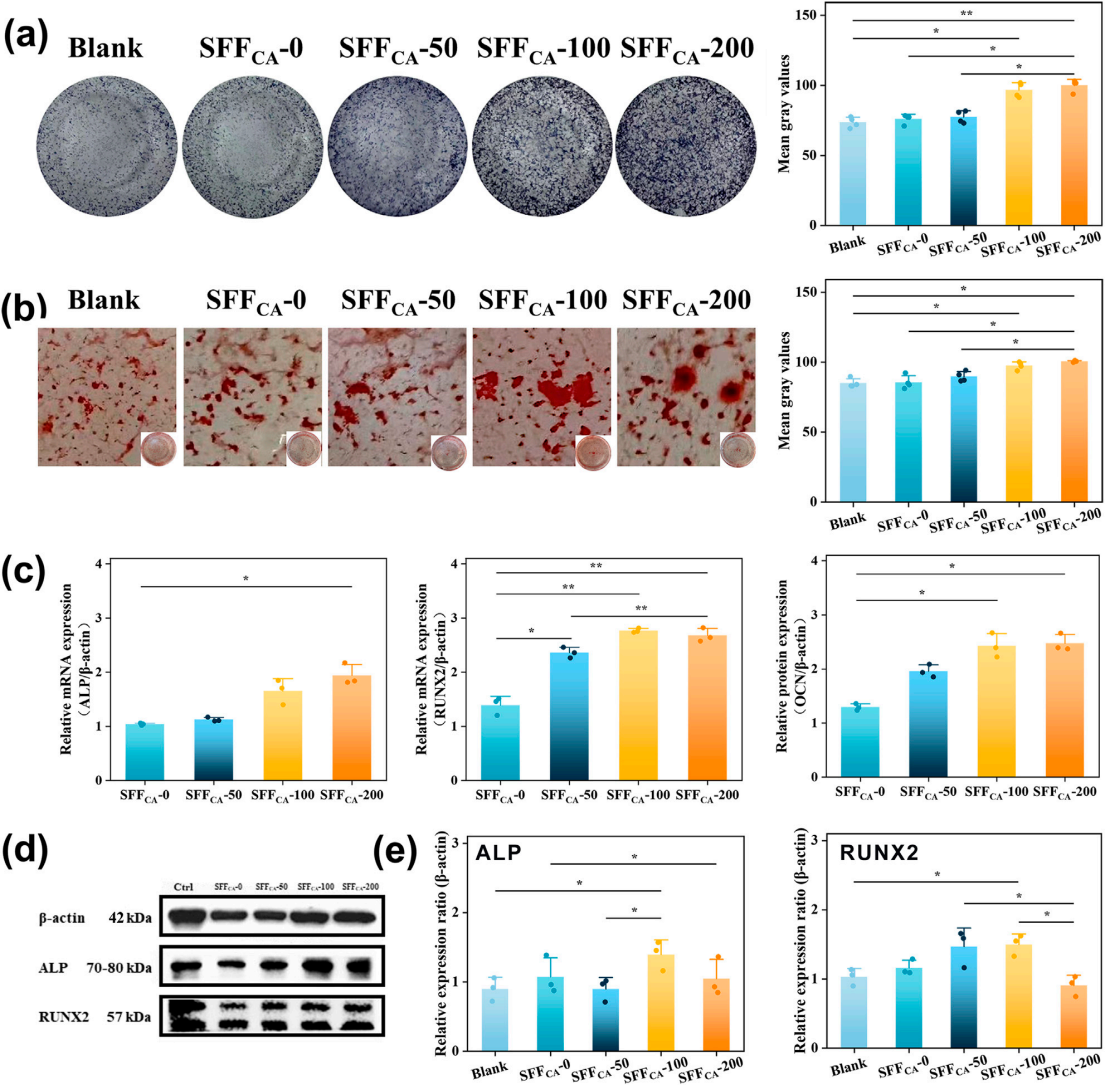
Osteogenic activity of SFF_CA_-IONPs **(a)** Corresponding staining area of ALP activity of rBMSCs incubated in cultured in the leach liquor of SF/IONPs composites for 14 days and **(b)** matrix mineralization for 28 days. The area of ALP activity and matrix mineralization was quantified using Image Lab software to determine the mean grey value for each image. **(c)** Expression of osteogenesis-related genes of MC3T3-E1 cultured in the leach liquor of SF/IONPs composites for 72 h. **(d)** Western blot shows protein levels of ALP, RUNX2 phosphorylated forms MC3T3-E1 cultured in the leach liquor of SFF_CA_-IONPs for 72 h. **(e)** Relative protein levels were quantified using Image Lab software. β-Actin was used as normalizer. Data are mean ± SD. Friedman’s test followed by corrected Dunn’s test were performed. Asterisk indicates statistically significant differences between control and experimental group (*: P < 0.05; **: P < 0.01).

Orthopedic scaffolds play a pivotal role in promoting the differentiation of osteoblasts into osteocytes through the incorporation of bioactive factors and the provision of structural and mechanical support (Singh et al., 2023). Natural polypeptide materials such as collagen and SF closely mimic the extracellular matrix (ECM), creating a microenvironment conducive to cell growth and differentiation (Xing et al., 2023). Compared to collagen, Silk fiber exhibits superior properties, including non-immunogenicity, controlled degradation, and excellent plasticity (Sun et al., 2021). Previous studies, including those by Meinel et al., have demonstrated that bone marrow stromal cells (BMSCs) exhibit enhanced adhesion and proliferation on silk fibroin membranes compared to polystyrene or collagen substrates (Meinel et al., 2005). Furthermore, Bissoyi et al. identified the presence of Arg-Gly-Asp (RGD) sequences in SF, which have been shown to activate integrins through the FAK signaling pathway, thereby inducing alkaline phosphatase (ALP) expression, promoting cell adhesion, and enhancing osteogenic bioactivity (Bissoyi et al., 2017). The SF-based membrane engineered by Wildt et al. exhibits bidirectional bioactivity, facilitating both osteoclast-mediated bone resorption and osteoblast-driven mineralization (Wildt et al., 2022). Chen et al. engineered a SF membrane characterized by the co-existence of both silk II and silk I crystalline structures, demonstrating enhanced osteogenic differentiation potential and effective orthotopic bone regeneration (Chen et al., 2024). Recently, combining SF with other materials has been reported to improve surface roughness, thereby enhancing cell adhesion while retaining the biological activity of the incorporated materials (Li et al., 2023). The incorporation of IONPs into SF scaffolds significantly increased ALP expression and its nuclear localization. As illustrated in Figures 7a,c–e indicating cell commitment to osteogenesis. The IONPs were validated to facilitate RUNX2 nuclear translocation, thereby promoting osteogenic differentiation and mineralization (Wang et al., 2016). Notably, there was a progressive increase in the concentration of IONPs, which was accompanied by a significant upregulation of RUNX2 expression and enhanced calcium deposition, as evidenced by alizarin red staining (Figures 7b–e). These results suggest that the osteogenic potential of SF-loaded IONPs strengthens with increasing IONP concentrations, offering a promising avenue for BTE applications.

## 4 Conclusion

In this study, self-assembled SFF_CA_ composites were prepared through solution casting and solvent annealing, forming a stable, water-insoluble silk Ⅱ crystal structure at room temperature without the need for harsh conditions. The incorporated IONPs were uniformly distributed within the SFF_CA_ matrix and did not adversely affect its structural integrity. Instead, their inclusion enhanced the porosity, thermal stability, and mechanical properties of SFF_CA_. Notably, cell culture experiments demonstrated that the addition of IONPs significantly improved the capacity of SFF_CA_ to support cell proliferation. In conclusion, the SFF_CA_-IONPs developed in this study represent a promising advancement in the field of SF-based biomaterials. Their enhanced properties and biocompatibility underscore their potential for diverse applications in biomedicine, tissue engineering, cell culture, and related fields.

## Data Availability

The original contributions presented in the study are included in the article/Supplementary Material, further inquiries can be directed to the corresponding authors.
