# Supplementary material for: Preparing the functional biomaterial with osteogenic bioactivities by incorporating annealing pretreated silk fiber and iron oxide nanoparticles
Source: Front Bioeng Biotechnol. 2025 Apr 10;13:1584081. doi: 10.3389/fbioe.2025.1584081 (PMC12018372; doi:10.3389/fbioe.2025.1584081)
Supplement: Supplementary file 1 [file DataSheet1.docx]

**Supplement Materials**

Preparing the functional biomaterial with osteogenic bioactivities by incorporating annealing pretreated silk fiber and iron oxide nanoparticles

Peng Wang^1#^, Hengda Wang^2#^, Xucai Wang^2^, Jiayu Gu^3^, Caoxing Huang^2*^, Jianfei Sun^1*^

^1^ State Key Laboratory of Bioelectronics, Jiangsu Key Laboratory for Biomaterials and Devices, School of Biological Science and Medical Engineering, Southeast University, Nanjing 210009, China.

^2^ Co-Innovation Center for Efficient Processing and Utilization of Forest Resources, College of Chemical Engineering, Nanjing Forestry University, Nanjing 210037, China.

^3^ Jiangsu Institute of Metrology, Nanjing 210023, China.

^#^ Peng Wang and Hengda Wang contributed equally to this work, regarding as the first author.

*** Correspondence:**Caoxing Huang, [hcx@njfu.edu.cn](mailto:hcx@njfu.edu.cn); Jianfei Sun, [sunzaghi@seu.edu.cn](mailto:sunzaghi@seu.edu.cn).

**Table S1** RNA primer sequences

| **Primer name** | **Gene sequence(5'to3')** |
| --- | --- |
| ALP-mice-F | CCAACTCTTTTGTGCCAGAGA |
| ALP-mice-R | GGCTACATTGGTGTTGAGCTTTT |
| OCN-mice-F | GCAATAAGGTAGTGAACAGACTCC |
| OCN-mice-R | CCATAGATGCGTTTGTAGGCGG |
| Runx2-mice-F | GACTGTGGTTACCGTCATGGC |
| Runx2-mice-R | ACTTGGTTTTTCATAACAGCGGA |
| B-actin-mice-F | GGCTGTATTCCCCTCCATCG |
| B-actin-mice-R | CCAGTTGGTAACAATGCCATGT |
